# Advances in Aqueous Electrolyte Engineering: From Fundamental Limitations to Emerging Strategies

**DOI:** 10.3390/molecules31203721

**Published:** 2026-10-16

**Authors:** Yong-Gang Sun, Yi-Han Zhang, Jian Xiong, Jin-Yi Ding, Yu Hu, Xin Wang, Jun-Yu Zhao, Hao-Jie Fan, Song Chen

**Affiliations:** 1 School of Chemistry and Chemical Engineering, Yancheng Institute of Technology, Yancheng 224051, China; sunyg86@iccas.ac.cn (Y.-G.S.); xiongjian1917@163.com (J.X.); djy11062026@163.com (J.-Y.D.); huyu@stu.ycit.edu.cn (Y.H.); wang92643864@163.com (X.W.); zjy19551502383@163.com (J.-Y.Z.); fhj18012096918@163.com (H.-J.F.); 2 Jiangsu Key Laboratory of Coastal Photovoltaic Technology and System, Yancheng Institute of Technology, Yancheng 224051, China

**Keywords:** aqueous electrolyte engineering, solvation structure, solid electrolyte interphase, water-in-salt electrolyte, deep eutectic electrolyte, molecular crowding, electrochemical stability window

## Abstract

Aqueous rechargeable batteries are promising candidates for large-scale energy storage owing to their inherent safety, low cost, and environmental compatibility, yet the narrow 1.23 V electrochemical stability window (ESW) of water limits their energy density. Since the seminal 2015 report of water-in-salt electrolytes (WiSE), electrolyte engineering—manipulating the molecular environment of water, including its O–H bond strength, hydrogen-bond topology, and interfacial reactivity—has emerged as the central strategy for overcoming this thermodynamic constraint. This review synthesizes the representative studies across Li^+^, Na^+^, K^+^, and Zn^2+^ chemistries into a unified framework that links the physicochemical fundamentals of aqueous electrolytes (ion solvation, hydrogen-bond networks, the electrical double layer, and ion transport) with five classes of strategies—concentration, solvation-structure, and interface engineering; emerging electrolyte systems (deep eutectic, ionic-liquid–water, asymmetric decoupled, gel, and high-entropy); and practical challenges (wide-temperature operation, cost, and scalability). Examining unresolved controversies—the thermodynamic versus kinetic origin of ESW expansion in WiSE, the long-term chemical persistence of aqueous solid electrolyte interphases, and the definitional boundary of aqueous batteries—this review proposes a research roadmap spanning multi-strategy orthogonal integration and standardized evaluation protocols, providing both a tutorial for newcomers and a critical reference for researchers pursuing practical high-voltage aqueous batteries.

## 1. Introduction and Fundamentals

Lithium-ion batteries (LIBs) have penetrated portable electronics, electric vehicles, and grid storage since their commercialization in 1991 [1,2,3,4]. Their reliance on LiPF_6_^−^ containing flammable organic carbonate electrolytes, however, poses intrinsic safety risks [5,6,7]. Thermal runaway, fires, and explosions have recurred across multiple catastrophic incidents [5,8,9,10]. Water—Earth’s most abundant, cheapest, non-toxic, and completely non-flammable solvent—has thus re-emerged as a core candidate for next-generation electrolyte design [11,12]. A comprehensive roadmap for the aqueous battery renaissance was laid out by Chao et al., who noted that aqueous batteries compete asymmetrically with organic lithium batteries through absolute safety, extreme low cost, and unlimited sustainability [13].

The key breakthrough came in 2015. Suo et al. reported a “water-in-salt” electrolyte (WiSE) composed of 21 m (mol kg^−1^) LiTFSI in Science, expanding the electrochemical stability window (ESW) of aqueous electrolytes from ~1.23 V to ~3.0 V for the first time [11]. At this ultra-high concentration, the H_2_O-to-Li^+^ molar ratio drops to ~2.6:1. Free water molecules nearly vanish. TFSI^−^ anions are forced into the primary solvation shell of Li^+^, and their preferential reductive decomposition generates a dense LiF solid electrolyte interphase (SEI)—the first effective SEI in aqueous battery history [14]. This dual mechanism—thermodynamic water activity suppression and kinetic SEI protection—ignited a decade of intense research.

Yet the hidden costs of the WiSE route were sharply quantified by Xie et al. Fluorinated lithium salt concentrations of 21–55 m mean the salt procurement cost alone could exceed the material cost of an entire lead-acid battery. Meanwhile, the environmental persistence of per- and polyfluoroalkyl substances (PFAS) has prompted the European Chemicals Agency (ECHA) to propose a legislative restriction in 2023 [15]. Separately, a fundamental chemical contradiction was pointed out by Li et al.; the Zn^2+^/Zn reduction potential (−0.76 V vs. SHE) lies below the hydrogen evolution reaction (HER) potential, making HER thermodynamically favorable, while MnO_2_/Mn^2+^ deposition–dissolution chemistry requires proton participation—optimally under acidic conditions—which aggravates Zn corrosion [16]. The hydration energetics baseline of Li^+^, Na^+^, and K^+^, together with the voltage ceiling imposed by water, was systematically documented by Lu et al.—a ceiling that has imprisoned aqueous batteries for nearly 150 years [17].

Under standard conditions (25 °C, pH = 0), water decomposes thermodynamically at merely 1.23 V—hydrogen evolution reaction (HER) at the anode and oxygen evolution reaction (OER) at the cathode—as established in Pourbaix’s classic atlas [18]. Thermodynamics alone, however, cannot explain why WiSE remains stable at ~3.0 V. The key conceptual distinction is: thermodynamic stability is governed by the Nernst equilibrium potential, whereas kinetic stability is determined by the activation barriers of the Volmer–Heyrovsky–Tafel elementary steps. Li et al. provided a complete description of HER elementary steps; under alkaline or neutral conditions, the Volmer step requires water dissociation (H_2_O + e^−^ → H^+^ + OH^−^), incurring an additional O–H bond-breaking barrier of ~0.76 eV, making neutral/alkaline HER kinetics 2–3 orders of magnitude slower than acidic HER [19].

**Figure 1:**
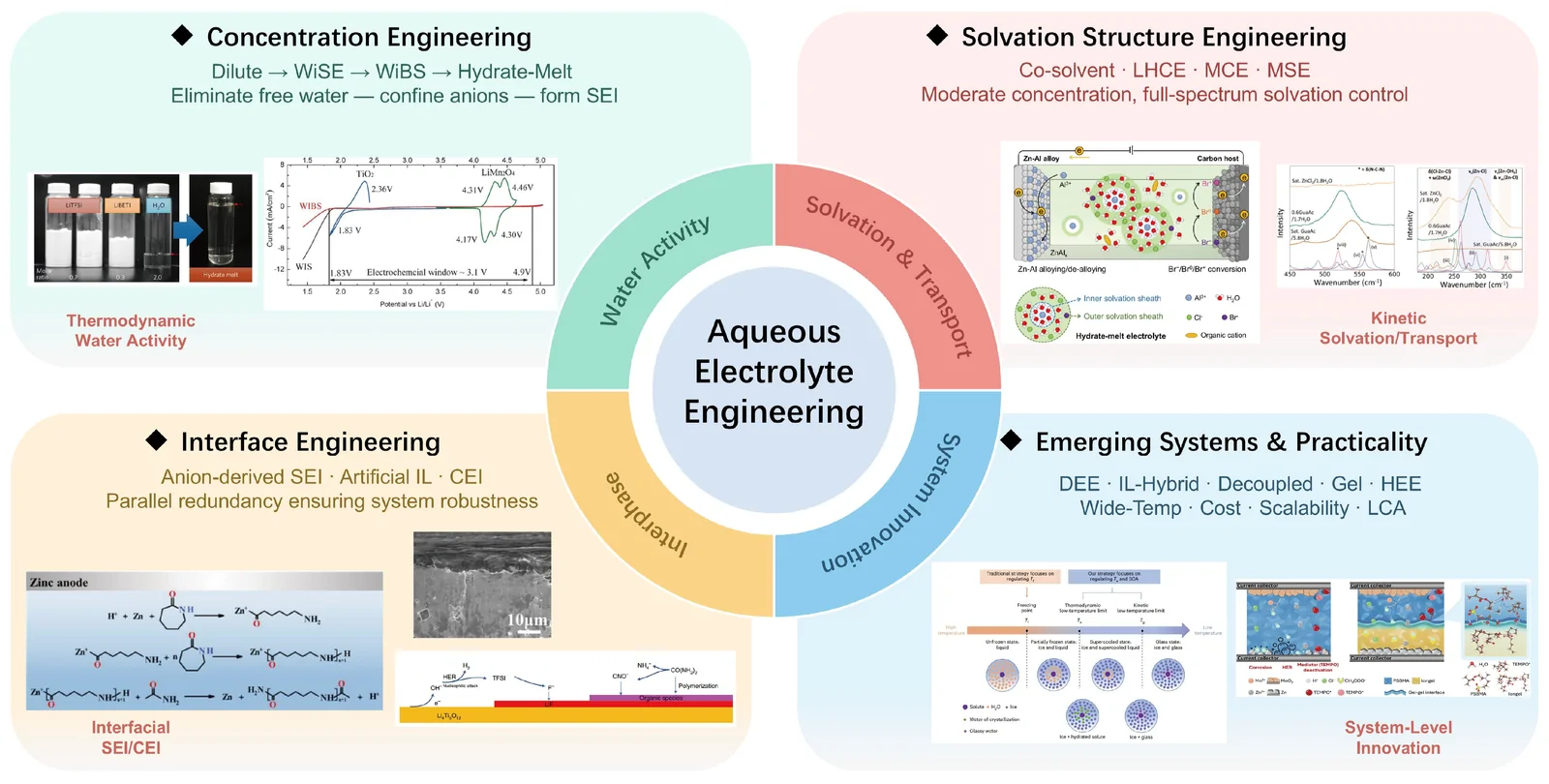
Schematic illustration of aqueous electrolyte tuning dimensions.

A deeper question is why the O–H covalent bond strength is not a fixed constant but a tunable variable. Vibrational spectroscopy by Perakis et al. showed that, when water molecules bind via O–H···O hydrogen bonds, electron donation from the acceptor oxygen weakens the donor O–H bond, manifesting as a redshift in stretching frequency (from ~3650 cm^−1^ to ~3200 cm^−1^) [20]. Any intervention that reduces water–water hydrogen-bond density will therefore strengthen residual O–H bonds and raise HER overpotential. In conventional 0.5–2 M Zn electrolytes, the Zn(H_2_O)_6_^2+^ hexaaqua complex was found to dominate, with Zn^2+^ coordination further weakening the O–H bonds of coordinated water molecules [21]. The combined effect—weakened O–H bonds plus ~55 M free water as an inexhaustible reactant reservoir—locks the CE of dilute Zn electrolytes at 70–85%, far below the >99.5% required for practical applications. Figure 1 summarizes the panoramic view of aqueous electrolyte tuning dimensions.

### 1.1. Ion Solvation Theory

The Born model—hydration free energy proportional to z^2^/r—provides the first quantitative framework for ion solvation, explaining why Al^3+^ is far more difficult to tune than Na^+^ [22,23]. The Born model describes only hydration strength, however, not the specific coordination structure of hydrated ions [24,25]. Comprehensive hydration number benchmarks—Li^+^ ~4, Na^+^ ~5–6, K^+^ ~6–8, and Zn^2+^ strictly 6 (octahedral Zn(H_2_O)_6_^2+^)—were compiled by Marcus from X-ray/neutron diffraction and EXAFS measurements, providing indispensable experimental references for solvation studies across the field [26].

These hydration numbers reflect static coordination in dilute solutions [27,28,29]. When the view is extended across the full concentration axis, solvation structures undergo a concentration-dependent dynamic evolution: SSIP → CIP → AGG [30,31]. In dilute solutions (<1 m), solvent-separated ion pairs (SSIP) dominate [30]. At intermediate concentrations (~1–5 m), anions partially displace water molecules to form contact ion pairs (CIP). At extremely high concentrations (>10 m), three-dimensional ion aggregate networks (AGG) develop [30,32]. Through MD simulations of the electrical double layer at electrode/electrolyte interfaces [33], the cross-system transferability of this spectrum was verified, providing the critical physical picture for subsequent discussions of interfacial SEI formation [30,34,35]. Inferences from theoretical simulations ultimately require direct experimental evidence. Exactly such evidence came from the hydrated eutectic electrolyte reported by Yang et al. In the Zn(ClO_4_)_2_·6H_2_O–succinonitrile system, the splitting of the ClO_4_^−^ symmetric stretching vibration peak in Raman spectra was assigned to the formation of Zn^2+^–ClO_4_^−^ direct contact ion pairs, becoming the spectroscopic fingerprint for CIP identification in the field [36].

The recurring concept of “free water” in the above discussions received precise thermodynamic clarification from Li et al. Through DSC, water in hydrogels was finely distinguished into three categories: non-freezable bound water, freezable bound water, and free water [37]. This trichotomy provides a unified language for understanding “water existence states” throughout this review. Building on this, the Hofmeister series—kosmotropic ions (Zn^2+^, Li^+^, Ac^−^) versus chaotropic ions (Gua^+^, I^−^)—was introduced into the design context of aqueous Zn electrolytes, providing key conceptual tools for the weakly solvating strategies discussed later [21].

We emphasize that “free water” is an operationally defined descriptor rather than a universally transferable physical quantity. Its measured value depends on the technique employed—differential scanning calorimetry (which distinguishes non-freezable bound, freezable bound, and free water), Raman/IR spectroscopy (O–H stretching frequency), or NMR—and quantitative cross-study comparisons therefore require the measurement method to be specified explicitly. Throughout this review, the term is used in this technique-specific sense.

### 1.2. Water Hydrogen-Bond Network: Topology and Disruption

In liquid water, each molecule participates in ~3.6 hydrogen bonds. The quantitative O–H frequency/hydrogen-bond strength correlation was established by Bakker and Skinner through ultrafast 2D-IR spectroscopy [38]. When salts dissolve, cation hydration and anion hydrogen-bond acceptance attack the tetrahedral hydrogen-bond network from two independent dimensions. MD simulations by Singh et al. quantified that the average number of free water–water hydrogen bonds decreases monotonically from ~3.6 to <1 as concentration increases from dilute to WiSE regimes [39].

Three independent experimental observations converge on a consistent principle. PEG crowding was shown to blueshift O–H stretching from 3200–3400 cm^−1^ to 3480 cm^−1^—weaker PEG–H_2_O hydrogen bonds replacing H_2_O–H_2_O hydrogen bonds [40]. The SL/DMSO/H_2_O ternary eutectic was demonstrated to disrupt the water–water hydrogen-bond network, reducing hydrogen-bond density and strengthening the residual O–H bonds (an O–H stretching blueshift), causing suppression of hydrogen evolution [41]. These findings jointly establish: O–H covalent bond strength is a direct function of its hydrogen-bond status—weaker/fewer hydrogen bonds mean stronger O–H bonds and higher overpotential required for electrochemical decomposition [42,43,44].

### 1.3. Electrical Double Layer and Electrochemical Stability Window

The electrode/electrolyte interface consists of the inner Helmholtz plane (IHP, directly adsorbed species), outer Helmholtz plane (OHP, fully solvated ions), and the diffuse layer [45]. A classic example was provided by Xu et al.; in 4.5 m LiTFSI–KOH–CO(NH_2_)_2_–H_2_O ternary eutectic, the LiF/polymer bilayer SEI shifted the cathodic limit from WiSE’s ~1.9 V by an additional ~400 mV to ~1.5 V vs. Li^+^/Li [46]. DMSO and SL co-solvents were further shown to block Grotthuss proton hopping at the cathode interface, creating a localized pH gradient that suppresses Zn corrosion [47].

It should be emphasized that an electrochemical stability window (ESW) is an operational quantity, not an intrinsic property of the electrolyte. The measured ESW depends on the working electrode (inert vs. active), scan rate, pH, and water activity; consequently, a single aqueous electrolyte cannot be assigned one universal ESW value. Every ESW cited in this review is therefore an operationally defined window measured under the specific conditions of the original study, and key values in Table 1 are annotated with their measurement conditions where reported.

### 1.4. Ion Transport Mechanisms

Ion diffusion in dilute solutions follows Stokes–Einstein behavior. In concentrated solutions, it was demonstrated that macroscopic viscosity severely underestimates actual diffusion coefficients, because ions migrate through low-density regions between aggregates [48]. The vehicular mechanism (ions translating with intact hydration shells) dominates monovalent and divalent cation transport [27,49,50]. The Grotthuss mechanism (protons hopping via sequential O–H bond breaking and forming through the hydrogen-bond network) is ~5–7× faster. Worth noting, any strategy that disrupts the continuous water hydrogen-bond network—WiSE, PEG crowding, and DES eutectics—suppresses HER through two independent pathways simultaneously: reducing free water thermodynamic activity AND severing Grotthuss proton transport channels to the anode surface.

To sum up, this review systematically covers representative research advances, spanning Li^+^ (ΔG_hyd_ = −475 kJ mol^−1^), Na^+^ (−365 kJ mol^−1^), K^+^ (−295 kJ mol^−1^), and Zn^2+^ (−1955 kJ mol^−1^) chemistries. Chapters are organized by electrolyte tuning dimensions—concentration (Section 2), solvation structure (Section 3), interfaces (Section 4), system paradigms and practical constraints (Section 5)—rather than by metal-ion chemistry, so cross-system mechanistic comparisons naturally emerge within each tuning dimension. Table 1 provides a comprehensive comparison of the representative electrolyte strategies discussed in this review. The unifying thesis is: water’s molecular environment—O–H bond strength, hydrogen-bond topology, and interfacial reactivity—can be engineered through molecular-level chemical intervention, transforming water from an immutable constraint into a programable design variable.

**Table 1:** Comprehensive comparison of representative aqueous electrolyte strategies.

Strategy	Representative System	Key Mechanism	Key Metric	Ref.
WiSE	21 m LiTFSI	Free H_2_O → 0; TFSI^−^ → LiF SEI	ESW ~3.0 V; CE > 99%	[11]
WiBS	21 m LiTFSI + 7 m LiOTf	TFSI^−^ + OTf^−^ co-decompose→ composite SEI	ESW ~3.1 V	[51]
Hydrate-Melt	Li(TFSI)_0.7_(BETI)_0.3_·2H_2_O	H_2_O: salt ≤ 3; no bulk water	ESW 3.0–3.8 V; CE > 99%	[36,52,53]
MCE	2 m LiTFSI + PEG (60–80 wt%)	PEG excluded volume replaces H_2_O H-bonds	ESW ~3.2 V	[40]
LHCE	LiTFSI–H_2_O–HFE (micelle-like)	Inert diluent → nanoclusters; viscosity ↓	Zn CE > 99.5%	[54,55]
Recessive Solvent	Zn(OTf)_2_ + HFIP; GuaAc/ZnCl_2_	2nd-shell bridging; DN ≈ 0	Zn DOD 88%; pouch 57 Wh kg^−1^	[21,56]
DEE	Acetamide–Caprolactam; SL/DMSO/H_2_O ternary	H_2_O as minority in eutectic network	Zn > 4000 h; CE > 97%	[47,57]
IL–H_2_O Hybrid	[BMIM]OTf/H_2_O	IL cations compete for H_2_O coordination	Zn CE > 99%	[58]
Asymmetric/Decoupled	Anolyte ≠ Catholyte	pH-decoupling: independent anolyte/catholyte	CE > 99.5%	[59,60,61]
Gel/ Quasi-Solid	WIPSE: PAM–LiTFSI–H_2_O	H_2_O confined in 3D polymer nanopores	ESW 3.2 V; 100% recyclable	[37,62]
High-Entropy	≥5 salts near-equimolar	ΔS_mix_ → single-phase; no crystallization	Ultra-low polarization	[63]
SEI/CEI	Sulfamate; PVDF-HFP/SiO_2_; SrTiO_3_	Low-LUMO anion → selective SEI	Zn > 2000 h; ESW 1.8 → 2.4 V	[64,65,66]
Additive	NAG;SCD;Glycerol	One molecule: solvation + SEI + pH	NAG: >4000 h, >2000 cyc SCD: Zn CE 99.8%	[15,67,68]
Anti-Freezing	DMF–KOH TMP–H_2_O–LiTFSI	T_e_/T_g_/σ framework; O–H strengthened ↓	<−82 °C operation	[19,69,70]

Note: “→” indicates a process or resulting effect, while “↓” indicates a decrease. Abbreviations: WiSE, water-in-salt; WiBS, water-in-bisalt; MCE, molecular crowding electrolyte; LHCE, localized high-concentration electrolyte; DEE, deep eutectic electrolyte; IL, ionic liquid; WIPSE, water-in-polymer salt electrolyte; SEI, solid electrolyte interphase; CEI, cathode electrolyte interphase; ESW, electrochemical stability window; CE, Coulombic efficiency; DOD, depth of discharge; DN, donor number; T_e_, eutectic temperature; T_g_, glass transition temperature; HFIP, hexafluoroisopropanol; HFE, hydrofluoroether; PAM, polyacrylamide; PEG, polyethylene glycol.

## 2. Concentration Engineering: From Dilute to Water-in-Salt to Hydrate-Melt

### 2.1. From the Dilute Era to Water-in-Salt Electrolytes: Discovery and Mechanistic Deconstruction

The aqueous LiMn_2_O_4_/VO_2_ battery demonstrated by Li et al. in 1994, confined to ~1.5 V, marked the birth of aqueous lithium batteries and their two-decade stagnation [71]. In 2 m LiTFSI dilute solutions, online electrochemical mass spectrometry (OEMS) directly detected uncontrollable H_2_ and O_2_ evolution [40]. In 0.5–2 M Zn electrolytes, Zn(H_2_O)_6_^2+^ dominating the IHP makes HER kinetically unavoidable [21]. The paradigm-shaping question confronting the field: can the existence state and chemical environment of water molecules be fundamentally altered by tuning salt concentration?

Suo et al.’s 21 m LiTFSI WiSE achieved ~3.0 V ESW by eliminating free water and forcing TFSI^−^ into the Li^+^ primary solvation shell. They argued that the LiF-rich interphase acts as an electronic barrier preventing water reduction while permitting Li^+^ migration. Evidence came from TEM images of cycled Mo_6_S_8_, revealing a 10–15 nm crystalline phase on the surface compared to pristine state [11]. The Li^+^ first-shell H_2_O coordination number was quantified as ~2.6, yielding a sobering conclusion: even pushing concentration to 63 m cannot lower this value below 2.0—there is a thermodynamic ceiling for simply piling up salt [46]. A theoretical framework was provided from ion aggregation thermodynamics: beyond a critical salt concentration, ions form an infinite percolation network, locking water molecules within ion hydration shells; the water chemical potential consequently decreases by orders of magnitude—thermodynamic suppression of HER, not merely kinetic [72]. The universality of this strategy was independently verified in non-aqueous systems [73]. Zn^2+^, however, faces far stronger anionic Coulombic constraints than Li^+^ due to its divalent charge, with a salt solubility ceiling far below that of Li systems [47].

### 2.2. Anion Chemistry and Multi-Salt Synergy: The Decisive Role of Salt Selection

**Figure 2:**
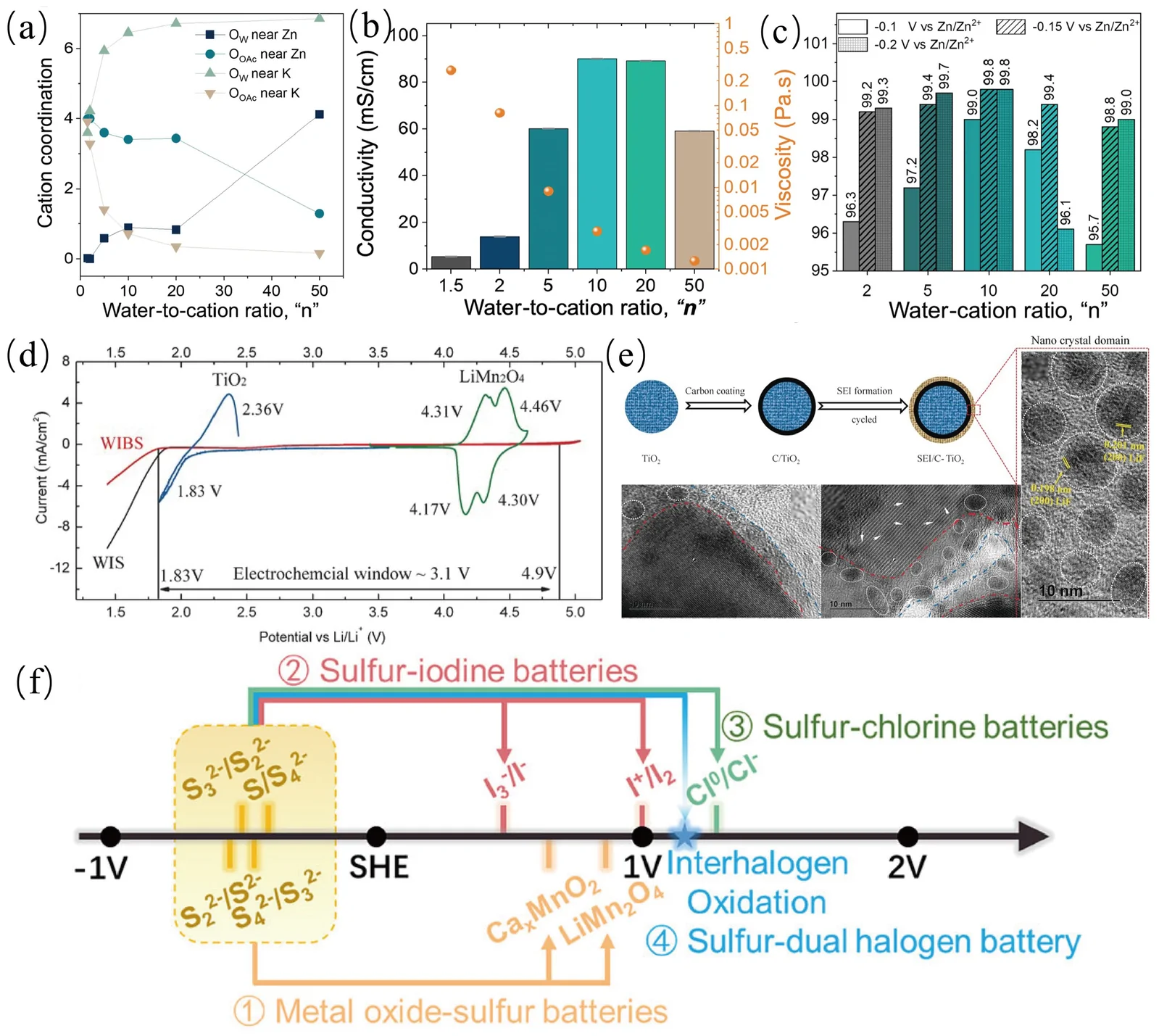
The Zn_0.2_K_0.8_OAc_1.2_·nH_2_O electrolyte series. (**a**) MD predicted coordination environment of Zn (within 2.5 Å) and K (within 3.6 Å) cations as a function of electrolyte concentration. (**b**) Averaged Coulombic efficiency as function of water-to-cation ratio “n” for the electrolyte series Zn_0.2_K_0.8_OAc_1.2_·nH_2_O. (**c**) Conductivity and viscosity as a function of water-to-cation ratio n [74]. (**d**) The cyclic voltammetry (CV) of electrolytes on different electrodes. The electrochemical windows of WiBS (21 m LiTFSI+7 m LiOTf) and WiSE (21 m LiTFSI) electrolyte as measured on inactive current collector (stainless steel grid) at scanning rate of 10 mV s^−1^, overlaid with the 1st CV traces collected on active anode (C-TiO_2_) and cathode (LiMn_2_O_4_) materials at scanning rate of 0.1 mV s^−1^ in the same electrolyte. (**e**) Post-mortem analysis on the cycled anatase C/TiO_2_ in water-in-bisalt electrolyte: schematic illustration of TiO_2_ surface pre-coated by carbon and in situ coated by SEI, and TEM images of C-TiO recovered after 10 cycles at 0.5 C [51]. (**f**) Schematic comparison of the redox potentials of different redox couples in different battery configurations, illustrating the sulfur-dual-halogen design [79]. Reproduced with permission from [51,74,79].

If concentration is the lever for tuning solvation structure, anion coordination ability is the core parameter determining lever amplification. A paradigm-overturning demonstration came from Gomez Vazquez et al.; Zn_0.2_K_0.8_OAc_1.2_·nH_2_O at only ~2 m total salt concentration—far below the WIS regime—achieved >99% CE and 10× higher conductivity than WiSE, with OAc^−^ actively entering the Zn^2+^ first shell (Figure 2a–c) [74]. This discovery directly shakes the dogma that “only high concentration can achieve low water activity”—what truly matters is what species occupies the Zn^2+^ first coordination sphere [75,76,77,78].

On the anion selection spectrum, TFSI^−^ and OTf^−^ are the protagonists of the WiSE era because of their strong charge delocalization and ability to form electrochemically stable fluorinated SEI (LiF/ZnF_2_) [51,80,81]. Critical scrutiny was, however, offered by Yang et al., noting that PFAS environmental persistence and ECHA’s 2023 legislative proposal will directly threaten the long-term viability of the fluorination route [15]. Concrete cost data were provided: industrial-grade PEG costs only 1/30–1/100 of battery-grade LiTFSI [40]. A third path was demonstrated by Yang et al.; traditionally “unstable” ClO_4_^−^ is effectively stabilized within the Zn(ClO_4_)_2_·6H_2_O–SN eutectic network—anion electrochemical stability depends on its solvation environment, not an intrinsic property of the molecular structure [36].

Building on this anion-centric perspective, the water-in-bisalt system—21 m LiTFSI–7 m LiOTf—was reported by Suo et al., pushing total salt concentration to 28 m and extending the ESW to ~3.1 V (Figure 2d), with dual-anion decomposition of TFSI^−^ and OTf^−^ producing a composite SEI (Figure 2e) [51]. In a subsequent application, this WiBS electrolyte enabled a highly reversible aqueous sulfur-dual-halogen battery (Figure 2f), where the Cl^−^-containing electrolyte stabilized the I^+^/I^0^ and Cl^−^/Cl^0^ redox chemistry through the formation of [ICl_x_]^1-x^ interhalogens, ultimately delivering a high energy density of 304 Wh kg^−1^ [79]. The multi-solute design was extended from the anion to the cation dimension—introducing high ionic potential auxiliary cations (Al^3+^, Ca^2+^) simultaneously lowers eutectic temperature (T_e_) and glass transition temperature (T_g_) [69]. Differences in monovalent ion hydration energetics were reviewed, noting that the divalent hydration energy of Zn^2+^ (−1955 kJ mol^−1^) makes displacing water by anions far more difficult than in Li^+^ systems [82].

### 2.3. Hydrate-Melt Electrolytes

**Figure 3:**
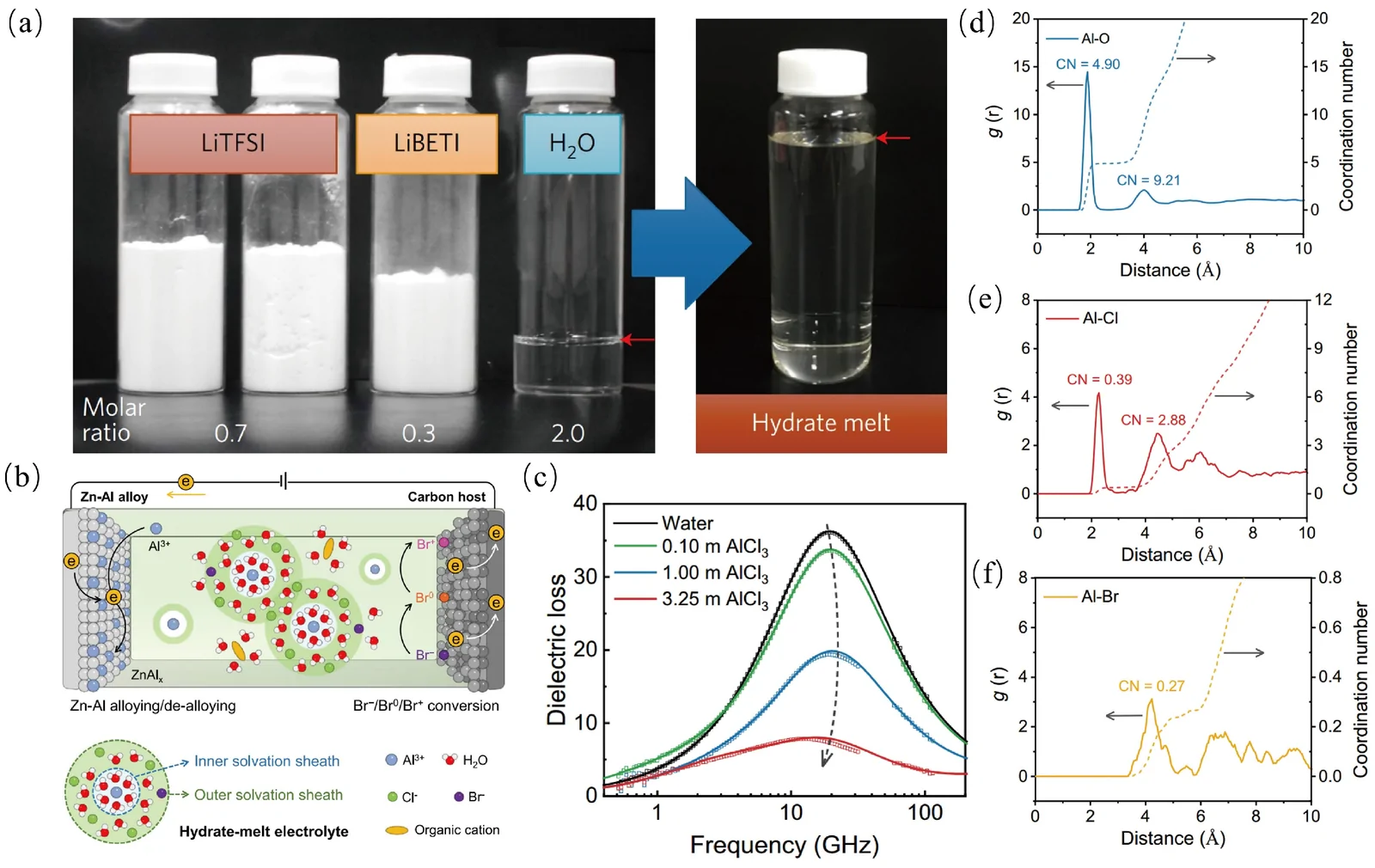
(**a**) Stoichiometric amounts of LiTFSI, LiBETI and water used to prepare a Li(TFSI)_0.7_(BETI)_0.3_·2H_2_O hydrate melt. The red arrows indicate the liquid levels [52]. (**b**) Schematic of an Al-Br battery enabled by a hydrate-melt electrolyte. The hydrate-melt electrolyte is featured by the predominant presence of water molecules engaged in ion solvation sheath and loosely bound halogen anions. (**c**) Dielectric loss spectra of aqueous AlCl_3_ solutions with varying concentrations, with corrections applied for contributions from the d.c. conductivity. Symbols correspond to experimental data and solid lines show the fitting curves. (**d**–**f**) RDFs (solid lines) and integral curves (dashed lines) of Al atoms extracted from AIMD simulations of 3.25 m AlCl_3_ + 1 m PY_14_Br, including (**d**) Al–O water, (**e**) Al–Cl, and (**f**) Al–Br [53]. Reproduced with permission from [52,53].

When salt concentration crosses a gradual threshold, the system transitions from “highly concentrated aqueous solution” to hydrate-melt—all water molecules participate in ion hydration shells, and bulk-free water ceases to exist. Yamada et al. first defined this concept with Li(TFSI)_0.7_(BETI)_0.3_·2H_2_O (H_2_O/Li^+^ = 2:1) (Figure 3a), where the water-ice phase transition peak in DSC completely disappeared and ESW reached ~3.8 V [52]. The most extreme experimental corroboration to date was provided by Chu et al.; in 3.25 m AlCl_3_ + 1 m PY_14_Br, AIMD-simulated radial distribution functions (RDFs) showed two solvation shells around Al^3+^, with Al–Cl distance 4.5 Å and Al–Br distance 4.2 Å, collectively indicating >98% of water molecules engaged in ion solvation (Figure 3b–f). Reversible Br^−^/Br^0^/Br^+^ conversion was achieved at 5 mAh cm^−2^ areal capacity with 267 Wh L^−1^ energy density—approaching the energy density of commercial lithium-ion batteries [53]. The precise boundary between hydrate-melt and highly concentrated WiSE (H_2_O/Li^+^ = 2:1 vs. 2.6:1, a difference of merely ~0.6 water molecules) remains an active controversy in the field.

WiSE and hydrate-melt succeeded because they indirectly achieved the right goals through extreme means (excess salt)—eliminating free water, confining anions, and forming SEI. They are limited because the means themselves are the source of the burden. Shen et al.’s review systematically quantified the intrinsic costs of high-concentration strategies across four dimensions: (i) viscosity increase by 1–3 orders of magnitude; (ii) ionic conductivity drop to ~10^−3^ S/cm; (iii) linear cost increase of fluorinated lithium salts; and (iv) performance collapse at low temperatures due to Arrhenius viscosity behavior and salt crystallization [83]. Cost data of 30–100× were also provided [40]. A rare case of recyclable design—80% LiTFSI recovery and polymer matrix regeneration—was demonstrated, but this remains an isolated effort in the field [62]. The paradigm-shifting question is as follows: can full-spectrum control of solvation structure be achieved through more precise chemical intervention without relying on excess salt?

## 3. Solvation Structure Engineering: Co-Solvents, Additives, and Moderate Solvation Strategies

The paradigm shift from concentration chemistry to solvation chemistry rests on one simple but profound physical fact: what determines interfacial chemistry is not the absolute salt concentration, but the species composition in the metal cation’s first coordination sphere, a solvation-first perspective echoed by recent electrolyte-engineering reviews across aqueous systems [84]. With PEG crowding at merely 2 m LiTFSI, an ESW of 3.2 V was achieved [40]. WIS-level performance was achieved at ~2 m from the anion coordination direction [74]. Three independently cooperable dimensions emerge: (i) ion–solvent affinity competition; (ii) hydrogen-bond network competition; and (iii) steric hindrance and confinement effects.

### 3.1. Organic Co-Solvent and Recessive Solvent Strategies

Gutmann donor number (DN) is the core parameter for co-solvent selection. A systematic comparison of DMSO (DN = 29.8), SL (DN = 14.8), and SN (DN = 15.0) revealed a fundamental trade-off: high-DN DMSO preferentially occupies the Zn^2+^ first shell but incurs a high desolvation energy barrier; low-DN SL achieves superior average CE (97%) but relatively shorter cycle life [47]. A complementary hydrogen-bond acceptor (HBA) strategy was demonstrated: SN disrupts water–water networks from the bulk perspective via hydrogen bonds with water, without entering the Zn^2+^ first coordination sphere [36]. Wang et al.’s acetamide–caprolactam DES electrolyte achieved functional division of labor between two organic components and generated an additional Zn^2+^-conductive organic protective layer on Zn surfaces through in situ electropolymerization [57]. The co-solvent logic was pushed to the intramolecular covalent bond level; DMF strengthens water’s O–H covalent bonds through DMF···H–O–H intermolecular hydrogen bonds, while KOH provides an alkaline environment to sever water dissociation; together, they achieve 4.3 V ESW and freeze-free operation below −82 °C [19].

**Figure 4:**
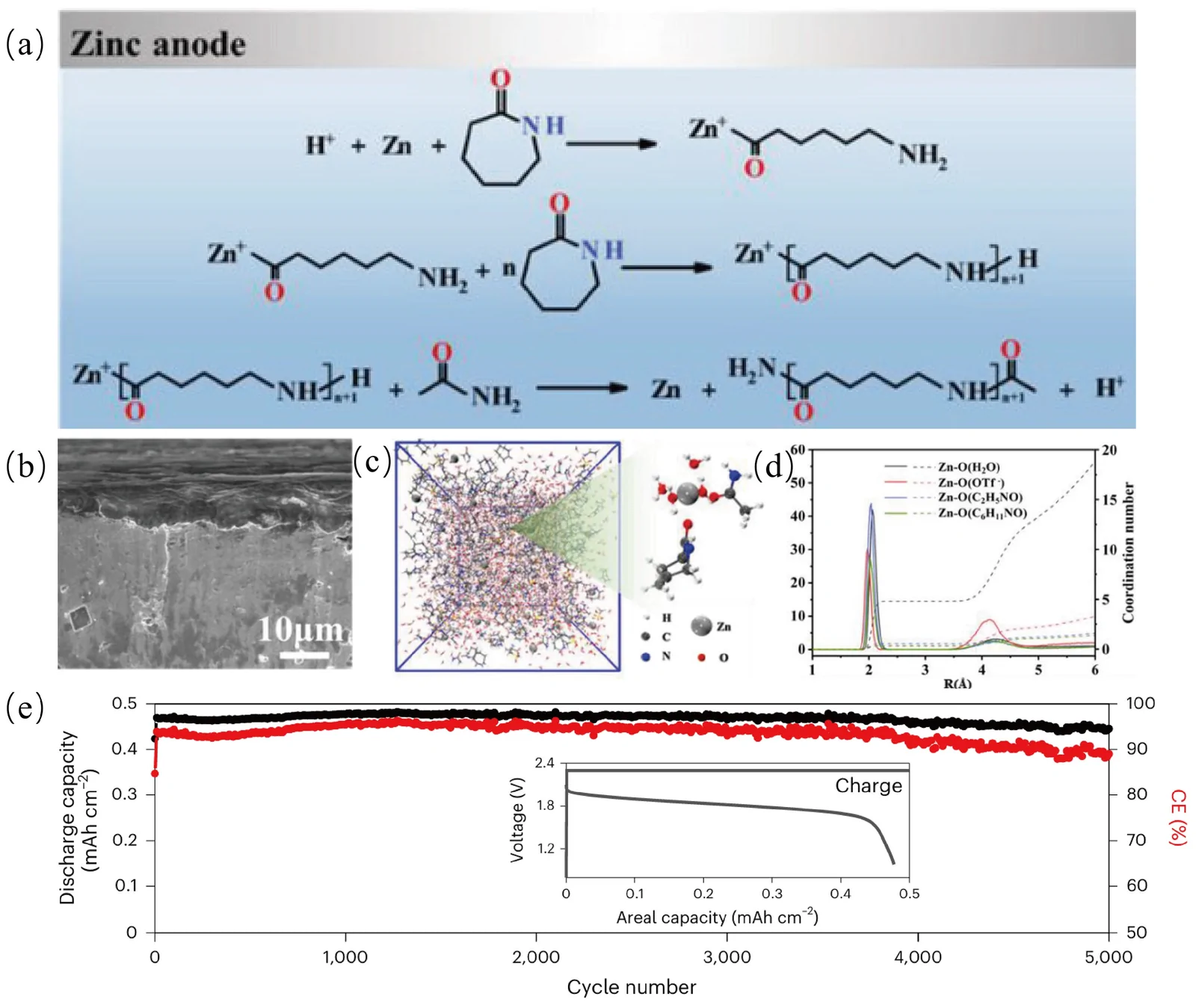
(**a**) Mechanism diagram of the amides polymerizing to the membrane on the anode surface. (**b**) 3D snapshot obtained by MD simulations and representative Zn^2+^-solvation structure of DES50. (**c**) RDFs for Zn–O(H_2_O), Zn–O(OTf^−^), Zn–O(C_2_H_5_NO), and Zn–O(C_6_H_11_NO) with a coordination number of Zn^2+^ from MD simulations. (**d**) Cross-profiles SEM images of Zn foils soaking in DES50 for 14 days at 25 °C [57]. (**e**) Voltage profiles and CE of full cells in SL/DMSO ternary electrolyte charged to 0.5 mAh cm^−2^ and discharged at 5 mA cm^−2^ [47]. Reproduced with permission from [47,57].

Another system-level strategy is the deep eutectic electrolyte (DEE), in which water is demoted from a host solvent to a tunable component within a multi-component eutectic network. Yang et al.’s Zn(ClO_4_)_2_·6H_2_O–SN hydrated eutectic pioneered the “ligand-assisted solvation” strategy, where SN participates in Zn^2+^ coordination through nitrile groups while disrupting water hydrogen-bond networks [36]. Wang et al.’s acetamide–caprolactam DES electrolyte provided additional protection through in situ electropolymerization on Zn surfaces (Figure 4a–c). Figure 4d shows cross-section SEM images of Zn foil after 14 days of immersion in DES50, displaying a uniform protective layer formed on the surface, in contrast to the corrosion morphology in DES0 [57].

Li et al.’s ternary SL/DMSO/H_2_O eutectic marks the transition of DEE from academic exploration to engineering validation. By integrating SL’s 97% average CE with DMSO’s 3500-cycle lifespan, they achieved 5000 cycles of stable operation at 95% average CE (Figure 4e) [47]. Practical deployment of DEE, however, faces two genuine obstacles: viscosity 1–2 orders higher than dilute solutions and severely insufficient long-term (>1000 cycles) oxidation stability data for amide/sulfone components at high cathode potentials. A balanced assessment of the potential and limitations of urea-based DES was provided [85].

These strategies, however, all involve organic molecules that either directly coordinate with Zn^2+^ or strongly interact with water, inevitably introducing a trade-off between solvation stability and desolvation kinetics. To circumvent this trade-off, the entirely new concept of “recessive solvent” was proposed. Molecules like hexafluoroisopropanol (HFIP) cannot independently dissolve zinc salts or explicitly coordinate with Zn^2+^ but are “activated” in electrolytes containing polar OTf^−^, forming “recessive solvent–anion–H_2_O” bridging structures that self-assemble into capsule-like nanoclusters encapsulating Zn(H_2_O)_6_ within [56]. The HFIP shell provides physical shielding without participating in Zn^2+^ coordination, so desolvation energy barriers are far lower than with strongly coordinating co-solvents like DMSO [86]. Symmetric cells achieved 3500 h of cycling at 50 mA cm^−2^ with 88.09% depth of discharge (DOD). Ah-level pouch cells achieved 57.14 Wh kg^−1^. Conceptually, this strategy expands the candidate molecular space from “solvents that can coordinate with Zn^2+^” to “all molecules that can be organized into solvation superstructures through anion assistance”.

### 3.2. Localized High-Concentration and Molecular Crowding Electrolytes

Localized High-Concentration (LHCE) introduces an inert diluent immiscible with salt but miscible with the host solvent, creating nanoscale high-concentration salt–solvent clusters in a low-viscosity continuous phase. The traditional model was fundamentally revised by Efaw et al. through SAXS and MD; clusters are not simple phase-separated “islands” but micelle-like structures with fuzzy interfaces and radial salt concentration gradients, with localization degree peaking near room temperature [54]. Three design insights are: (i) “localization” is a continuous attenuation gradient, not a binary criterion; (ii) there exists an optimal temperature window for SEI formation efficiency; (iii) diluent selection requires moderate cluster–interface interactions. These insights underscore that the effectiveness of LHCE relies on a delicate balance between cluster integrity and interfacial dynamics, rather than complete phase segregation.

Complementing this nanoscale cluster approach, the molecular crowding concept was pioneered by Xie et al., inspired by the biophysical phenomenon in living cells where macromolecules such as proteins and polysaccharides (>80 mg mL^−1^) dramatically reduce solvent water activity through excluded-volume effects [40]. With liquid PEG 400 as a crowding agent, 2 m LiTFSI–94%PEG–6%H_2_O achieved 3.2 V ESW, with OEMS detecting zero H_2_/O_2_/CO/CO_2_ signals. The LTO/LMO full cell maintained 75 Wh kg^−1^ after 300 cycles. Molecular crowding differs taxonomically from coordination competition (co-solvents) and ion confinement (WiSE); it uses topological exclusion of polymer chains to reduce water activity through purely physical means, fundamentally circumventing the side-reaction risks accompanying chemical intervention. Together, LHCE and molecular crowding strategies represent two non-coordinating routes—one operating at the nanoscale via salt–solvent clustering, the other at the macromolecular scale via excluded-volume effects—that achieve water activity suppression without strong coordination, thereby decoupling desolvation kinetics from thermodynamic stability.

### 3.3. Functional Small-Molecule Additives

Additive strategies—typically at 0.1 mM to 1 M—act simultaneously on the bulk, electrical double layer and electrode interface through multiple mechanisms. The unprecedented concept of liquid crystal interphase was introduced by Li et al.; merely 0.1 mM of non-ionic surfactant self-assembles into a gradient liquid crystal phase (lamellar → hexagonal → micellar) in conventional ZnSO_4_/MnSO_4_ electrolyte, synchronously templating preferential c-axis deposition of both Zn and MnO_2_. The dual-electrode-free cell achieved ~950 cycles with 80% capacity retention [87].

**Figure 5:**
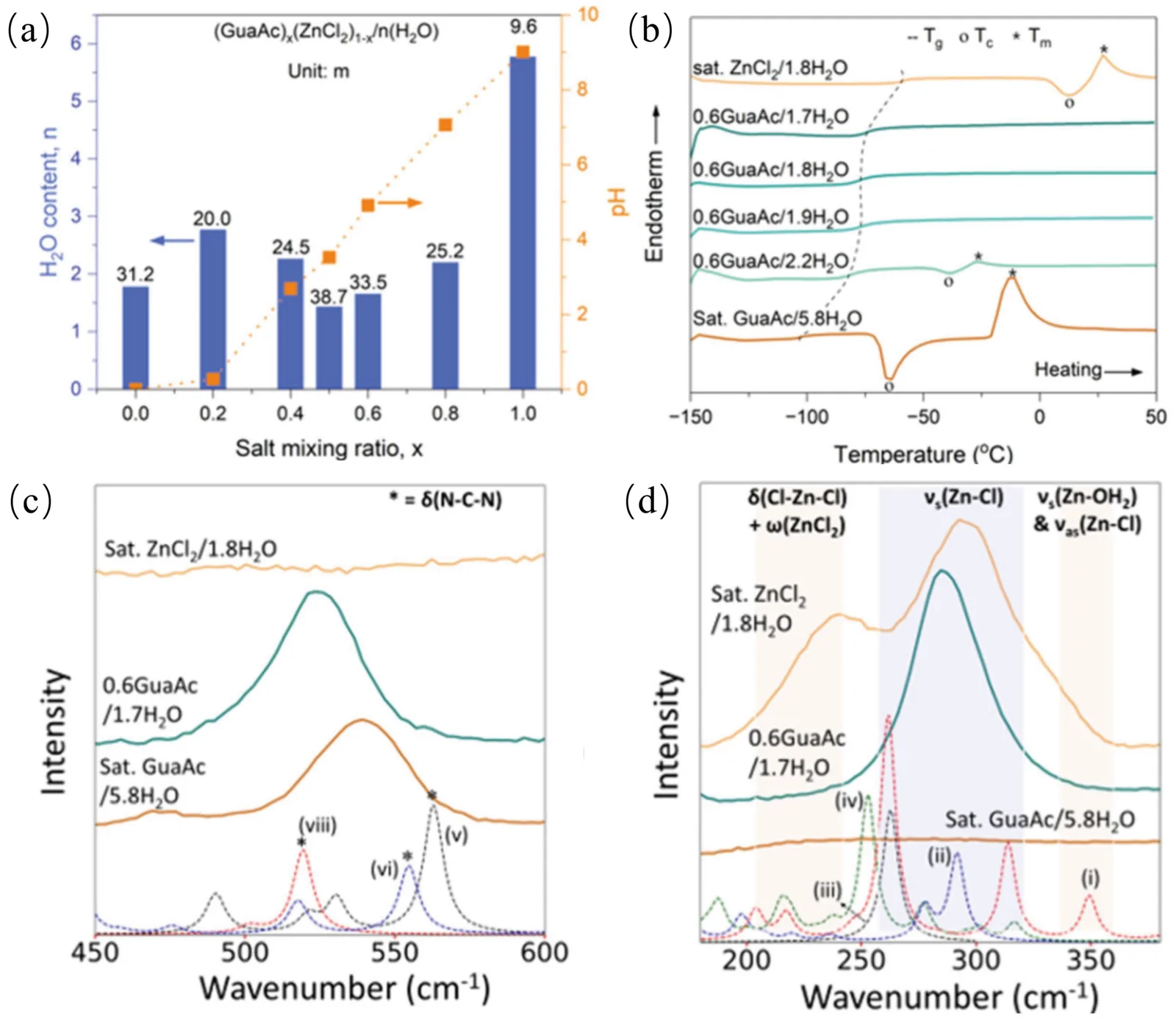
Compositional and local structure characterization of the GuaAc/ZnCl_2_ electrolytes. (**a**) Total salt concentration, water content, and pH of the chosen salt mixing ratios. (**b**) DSC analysis of the chosen electrolytes. (**c**,**d**) Experimental and calculated (dashed trace) Raman spectra corresponding to each species (reproduced with permission from [21]).

A sulfobutyl-β-cyclodextrin (SCD) supramolecular additive was deployed. Its densely packed outer sulfonate groups capture Zn^2+^ with zincophilicity, while the hydrophobic inner cavity repels water molecules and promotes desolvation. Zn symmetric cells operated stably for 3900 h at 2 mA cm^−2^ [67]. The Hofmeister series was used as a guide. Figure 5a shows the phase diagram of total salt concentration, water content, and pH as a function of composition in the GuaAc/ZnCl_2_ electrolyte system—a direct visual expression of how the relative ratio of kosmotropic Ac^−^ and chaotropic Gua^+^ precisely tailors electrolyte composition. Figure 5b–d demonstrates how GuaAc introduction alters the Zn^2+^ coordination environment, allowing anions (Cl^−^/Ac^−^) rather than water molecules into the first solvation shell—molecular-level evidence for “weakly solvating electrolytes” [21].

**Figure 6:**
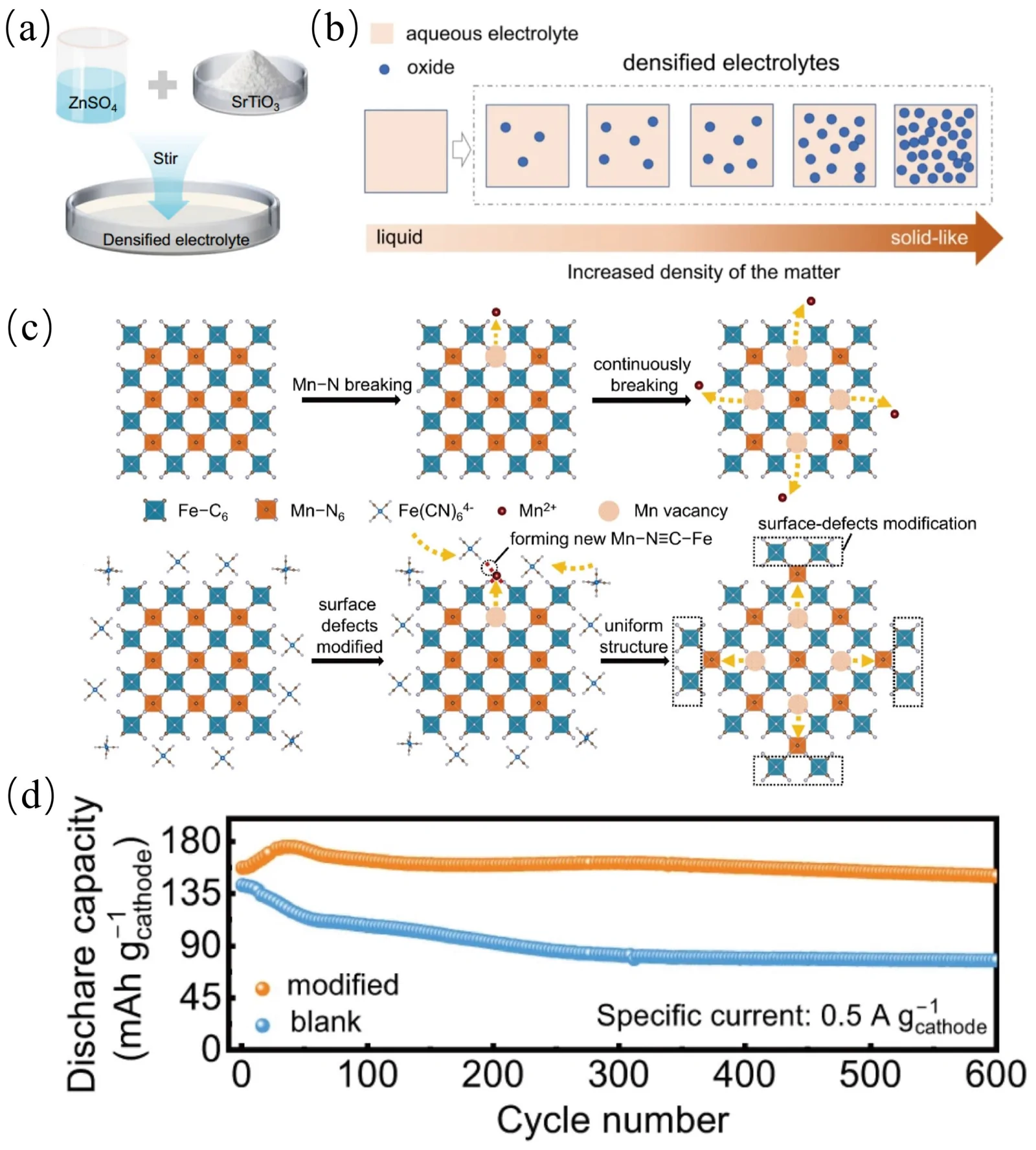
(**a**) Schematic diagram of densified aqueous electrolytes. (**b**) Schematic illustration of densified electrolytes formed by increasing density of solution after the addition of oxide [66]. (**c**) Schematic illustration of the Mn dissolution process. (**d**) Comparison of the cycling performance of PTCDI||NaFeMnF in the blank and modified electrolytes at 25 °C [88]. Reproduced with permission from [66,88].

A densified electrolyte was formed by mixing perovskite SrTiO_3_ nanopowder with 2 M ZnSO_4_ at a 1:1 mass ratio (Figure 6a,b). The oxide surface reduces water activity through weak hydrogen-bond adsorption, increases Zn^2+^ transference number, and induces (002) preferential deposition [66]. A “cation-trapping” strategy was proposed: Na_4_Fe(CN)_6_ as additive, with Fe(CN)_6_^4−^ capturing dissolved Mn^2+^ in situ and epitaxially filling surface vacancies (Figure 6c), enabling aqueous Na-ion batteries to retain 73.4% capacity after 15,000 cycles with 94 Wh kg^−1^ energy density (Figure 6d) [88].

Yang et al.’s N-acetyl-D-glucosamine (NAG) additive represents the “one molecule, multiple mechanisms” design philosophy. C=O/O–H groups modulate the solvation shell. NAG promotes SO_4_^2−^ catalytic decomposition to generate ZnS. Its own decomposition products form Zn chelates/ZnCO_3_ organic–inorganic hybrid SEI. Zn symmetric cells achieve 6500 h of stable cycling, completely fluorine-free [15].

## 4. Interface Engineering: SEI/CEI Construction and Regulation

**Figure 7:**
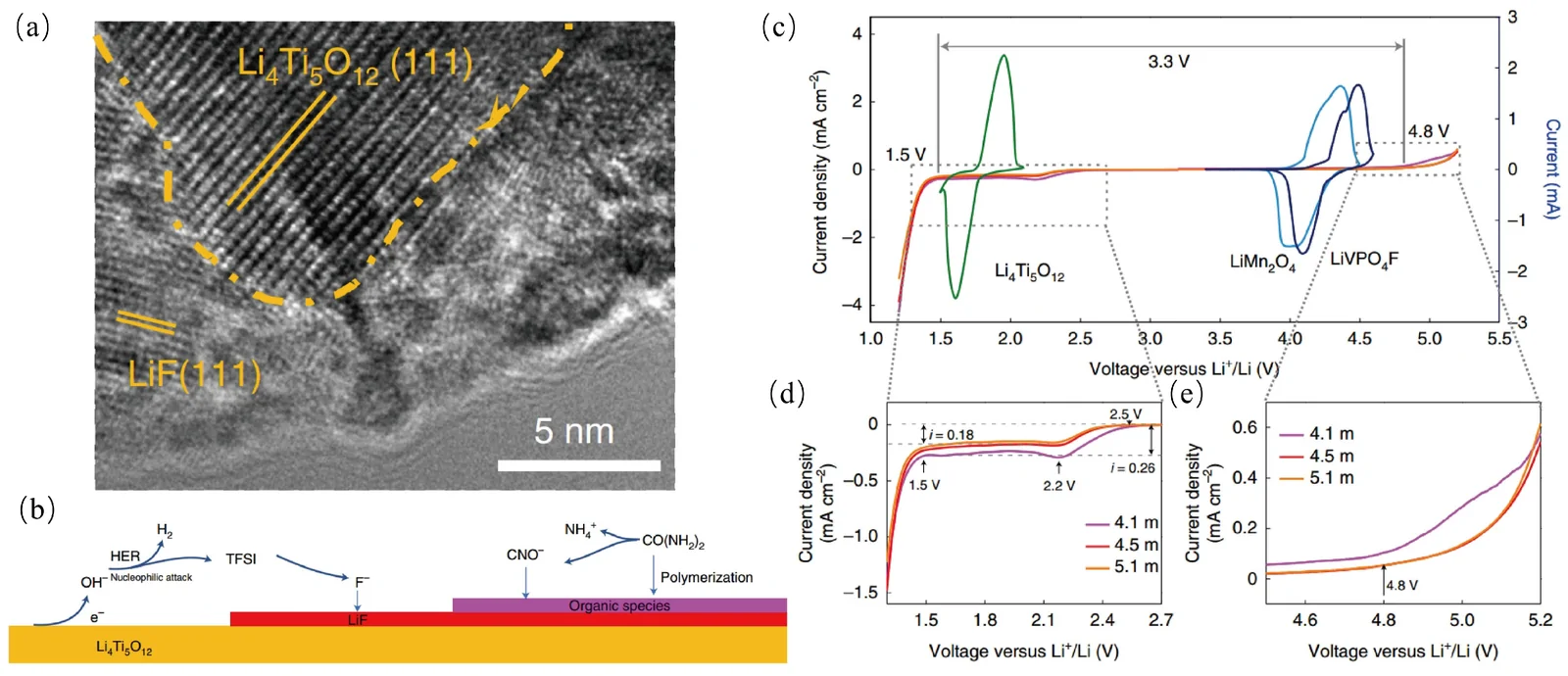
(**a**) High-resolution transmission electron microscopy images of cycled Li_4_Ti_5_O_12_ covered with a LiF/polymer bilayer SEI. (**b**) Schematic illustration of the formation of the inorganic/organic mixed SEI. HER, hydrogen evolution reaction. (**c**) Overall electrochemical stability window of the electrolytes with different LiTFSI concentrations, and redox of electrodes overlaid with cyclic voltammograms on current collectors. The redox currents of active electrodes (Li_4_Ti_5_O_12_, LiMn_2_O_4_ and LiVPO_4_F) in 4.5 m electrolytes were measured using a cyclic voltammogram at a scanning rate of 0.2 mV s^−1^. (**d**,**e**), The enlarged view of the regions outlined near the cathodic (**d**) and anodic (**e**) extremes (reproduced with permission from [46]).

The independent importance of interfacial chemistry emerges from the fact that the complete causal chain of the previously discussed solvation engineering strategies ultimately converges at the electrode/electrolyte interface. Here, three physical quantities absent in the bulk—electric field gradients exceeding 10^7^ V/cm, concentration gradients, and local pH gradients—nonlinearly couple to create a chemically distinct reaction environment. It was demonstrated that, in 4.5 m LiTFSI–KOH–CO(NH_2_)_2_–H_2_O, CO(NH_2_)_2_ co-reduces with LiTFSI at the interface under KOH catalysis to form a LiF/polymer bilayer SEI (Figure 7a,b). It is this interfacial reduction—not CO(NH_2_)_2_ hydrogen bonding with H_2_O in the bulk—that drives cathodic limit extension to ~1.5 V vs. Li^+^/Li (Figure 7c–e) [46]. DMSO and SL were revealed to act as “proton fences” at the cathode interface—capturing H^+^ released during MnO_2_ deposition and blocking Grotthuss proton hopping pathways. Interfacial chemistry is not an appendage of bulk solvation engineering; it is a parallel, independent design dimension.

### 4.1. Anion-Derived SEI: From Serendipity to Rational Design

The first rational SEI design principles were established. Replacing SO_4_^2−^ in conventional ZnSO_4_ with sulfamate (NH_2_SO_3_^−^)—which has a lower LUMO energy level and stronger Zn surface adsorption driving force, causing spontaneous enrichment at the IHP rather than OHP—leads to preferential reduction forming a sulfur-containing protective SEI, maintaining 99.65% average CE over 1000 cycles [64]. This proves that SEI formation is a function of the anion’s spatial position in the EDL, not its bulk concentration.

Universal design principles for organic/inorganic hybrid SEI were proposed: inorganic components (Sn alloy and NaCl) provide high Young’s modulus >39 GPa to resist dendrite puncture and fast ion diffusion pathways; organic aromatic components form dense elastic films to absorb volume fluctuations [89]. Comparative reviews have catalogued SEI construction strategies—electrolyte optimization, additives, and surface modification—across battery chemistries [90]. Yang et al.’s NAG fluorine-free hybrid SEI (Zn chelates/ZnS/ZnCO_3_, 6500 h) directly corroborates this principle in aqueous Zn systems [15].

A PVDF-HFP/SiO_2_/Zn(OTf)_2_ hybrid coating was designed to integrate three orthogonal functions—hydrophobic water-blocking, hydrophilic wetting, and Zn^2+^ conduction—in a single coating, with symmetric cells operating >960 h [65]. Eu^3+^-doped β-PVDF was reported as an artificial SEI: Eu^3+^ Lewis acid sites homogenize Zn nucleation spatial distribution, and the high-polarity β-PVDF matrix provides uniform local electric field, achieving 500 h of stable operation at 20 mA cm^−2^ [91]. AILs face three practical challenges: long-term adhesion under micron-scale Zn volume changes, the zero-sum trade-off between coating thickness and volumetric energy density, and additional manufacturing process complexity.

### 4.2. Cathode Electrolyte Interphase

Over 80% of interface studies focus on the anode side, yet cathode-side transition metal dissolution, structural phase transitions, and surface pH fluctuations are equally lethal. Liang et al.’s Na_4_Fe(CN)_6_ as an in situ CEI repair agent was discussed earlier. Yang et al.’s Zn(ClO_4_)_2_·6H_2_O–SN eutectic eliminates the driving force for organic cathode active material dissolution by abolishing bulk-free water—3500 cycles with merely 0.004%/cycle decay [36]. A PEG-Na^+^ surface complex on VHCF cathodes was identified through ^23^Na magic-angle-spinning (MAS) solid-state NMR—it forms through thermodynamically driven surface self-assembly, stabilizing surface Na^+^ sites on the open-framework structure with 90.3% retention after 2000 cycles [92]. No dedicated CEI combining universality and designability has been reported—this gap stems from the stringent oxidation stability requirements that high cathode potentials impose on organic components.

**Figure 8:**
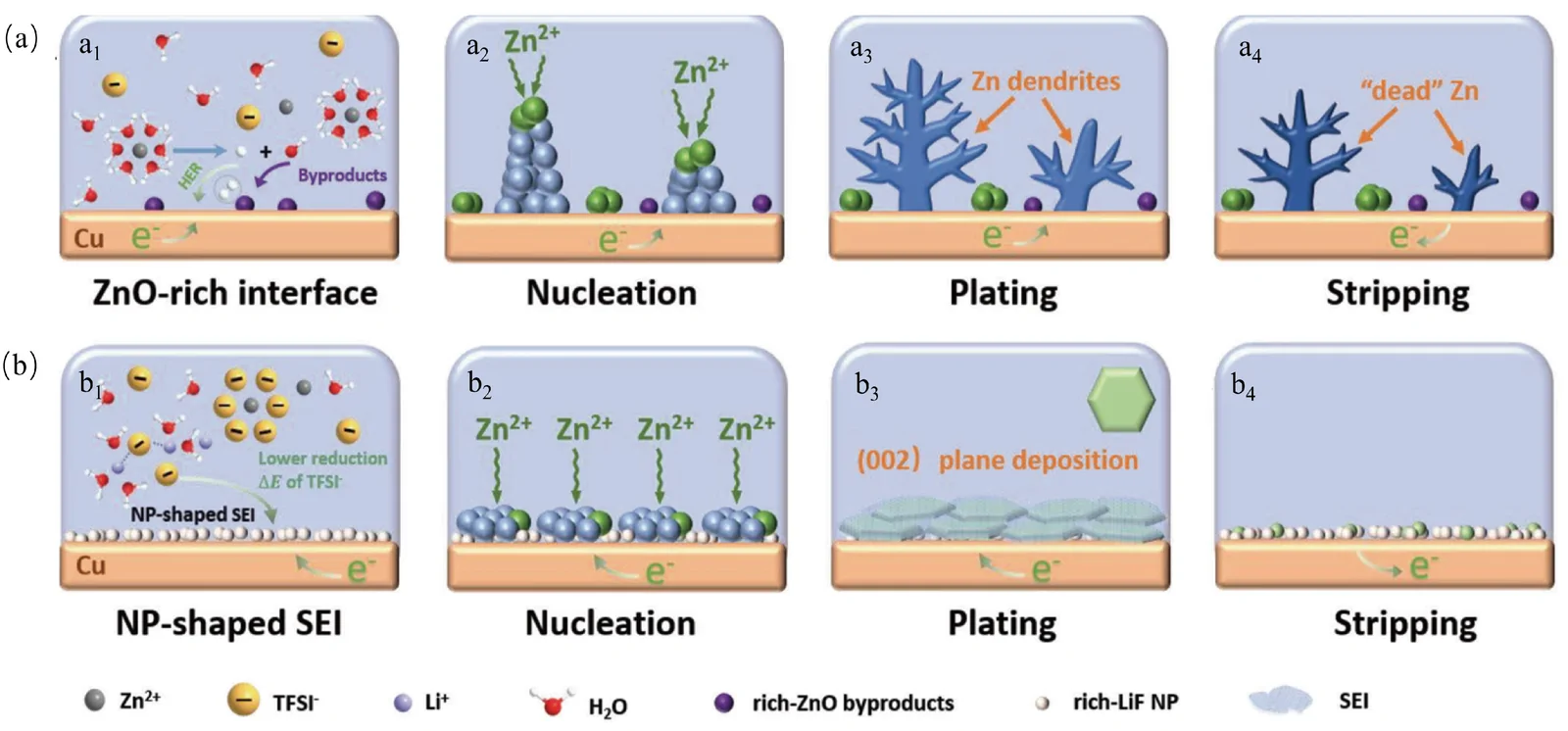
Interphasial formation and Zn plating/stripping at the interface in (**a**) 1 m Zn(TFSI)_2_ and (**b**) WIS systems. (**a_1_**) Formation of uneven ZnO-rich interface. (**a_2_**) Heterogeneous Zn nucleation. (**a_3_**) Growth of Zn dendrites. (**a_4_**) A large amount of “dead” Zn after stripping. (**b_1_**) Formation of uniform and compact LiF-rich NP-shaped SEI. (**b_2_**) Homogeneous Zn nucleation. (**b_3_**) Growth of hexagonal Zn (002) crystal plane. (**b_4_**) Even NPs after stripping (reproduced with permission from [93]).

While cathode-side interfacial design still lacks a universal and designable CEI, anode-side characterization has provided the most direct evidence for how interfacial chemistry governs SEI formation and deposition morphology. OEMS was used to directly monitor zero signals for H_2_ (*m*/*z* = 2), O_2_ (*m*/*z* = 32), CO (*m*/*z* = 28), and CO_2_ (*m*/*z* = 44) during 1C charge–discharge of LTO/LMO full cells—the strongest “negative evidence” for complete suppression of water decomposition [40]. In situ AFM combined with optical microscopy revealed that, in 1 m Zn(TFSI)_2_ + 20 m LiTFSI WiSE, a uniform, dense LiF-rich SEI forms before Zn deposition and induces hexagonal Zn growth along the (002) plane, while dilute solutions form thin, non-uniform ZnO-rich layers leading to randomly oriented dendrites (Figure 8a,b) [93]. Cryo-EM revealed that SEI nanostructure is highly sensitive to the electrolyte salt anion and is a critical performance-determining variable [94]. Fewer than 20% of published studies employ in situ interfacial characterization techniques. Successfully applying cryo-EM to aqueous battery SEI characterization will require overcoming the technical barrier that water vitrification is far more complex than in non-aqueous systems.

The comparatively slower development of cathode-side interphase engineering stems from three factors: (i) the stringent oxidative stability required of any organic CEI component at high cathode potentials, (ii) transition-metal dissolution and structural phase transitions that continually re-expose fresh surfaces [88], and (iii) surface pH fluctuation during (de)intercalation [47]. Concrete strategies that can address this gap include in situ CEI repair through electrolyte additives (e.g., Na_4_Fe(CN)_6_, which captures dissolved Mn^2+^ and epitaxially fills surface vacancies) [88], stabilization of open-framework cathode surfaces by polymer–Na^+^ surface complexes (e.g., the PEG–Na^+^ interface on VHCF) [92], and hydrated-eutectic electrolytes that abolish bulk-free water to suppress cathode active-material dissolution [36].

## 5. Emerging Systems and Practicality: From Novel Electrolyte Architectures to Real-World Constraints

### 5.1. Ionic Liquid–Water Hybrid Electrolytes

**Figure 9:**
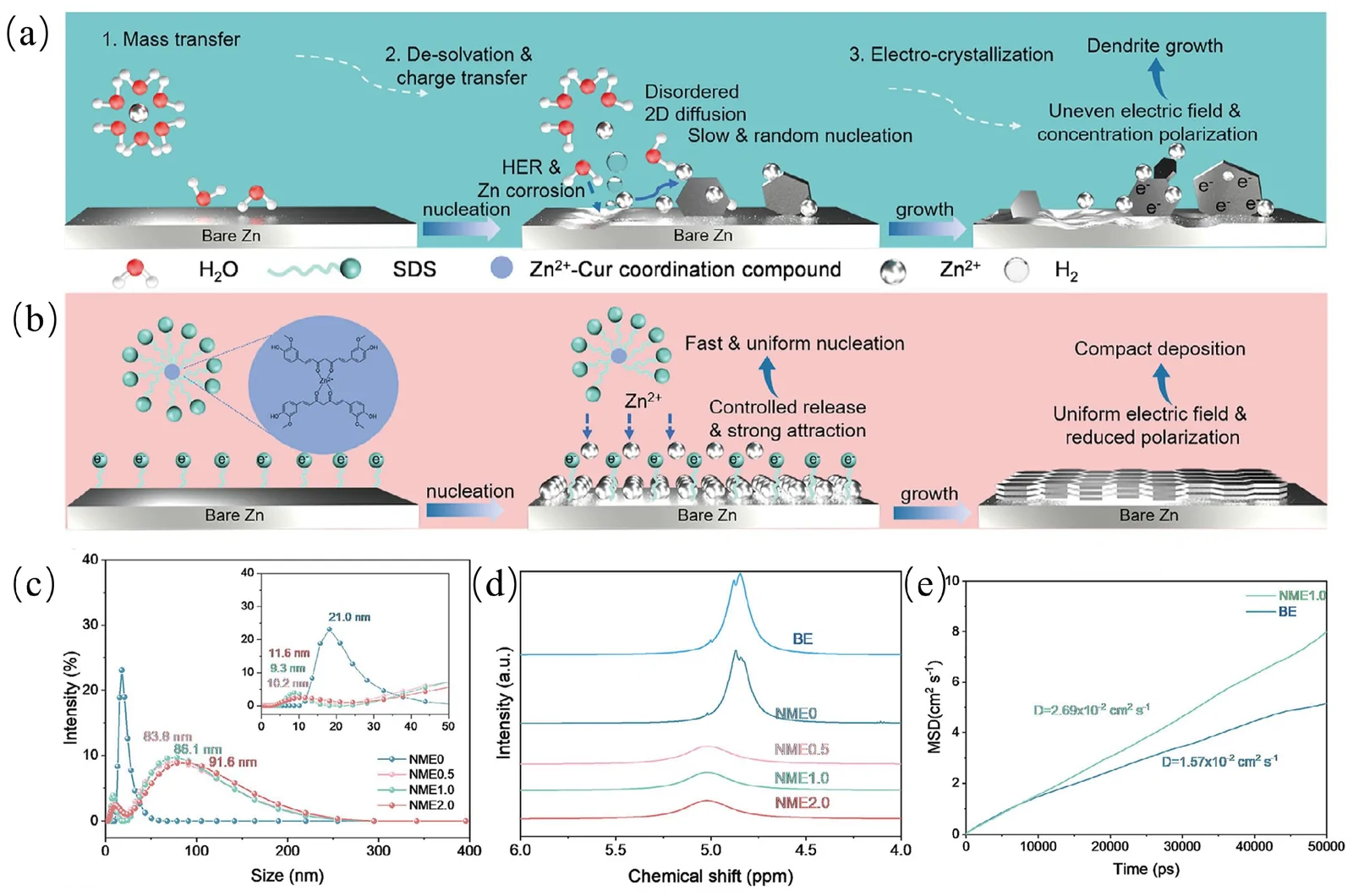
Regulation of Zn^2+^ solvation structure and transfer kinetics. Diagram of the nucleation and growth behavior of Zn^2+^ in (**a**) BE and (**b**) NME 1.0 electrolytes. (**c**) DLS analysis for the nanomicelles size distribution of different electrolytes. (**d**) ^1^H chemical shift of different electrolytes. In situ FTIR spectroscopy of Zn plating process performed at 5 mA cm^−2^. (**e**) The mean square displacement (MSD) and diffusion coefficient (D) of Zn^2+^ in different electrolytes (reproduced with permission from [55]).

Ionic liquid–water (IL-water) hybrid systems shift the chemical essence of the electrolyte from “salt dissolved in molecular solvent” to “mutual dissolution of two ionic media—water and IL.” Liu et al.’s choline chloride–urea DES/water mixture for Zn/Prussian blue batteries first verified that bulky IL cations improve Zn deposition morphology through steric hindrance [58]. Zhang et al.’s SDS–curcumin nanomicellar electrolyte pushed the IL-water logic to supramolecular self-assembly. DLS confirmed that curcumin encapsulation expanded the SDS micelles from ~21 to ~84–92 nm (Figure 9c), confining Zn^2+^ within micellar cores and imposing an ordered, symmetric nucleation/growth pathway versus the random deposition in the bare SDS electrolyte (Figure 9a,b). The ^1^H NMR downfield shift of the water peak further evidenced disruption of the continuous H-bond network (Figure 9d), while MD-derived mean-squared displacements showed enhanced Zn^2+^ diffusivity (Figure 9e), together rationalizing how the micelles physically interrupt Grotthuss proton hopping and enable controlled Zn^2+^ release [55]. DEE and IL-water occupy complementary ecological niches; the most promising forward path may be their hybridization.

### 5.2. Gel and Quasi-Solid-State Electrolytes

**Figure 10:**
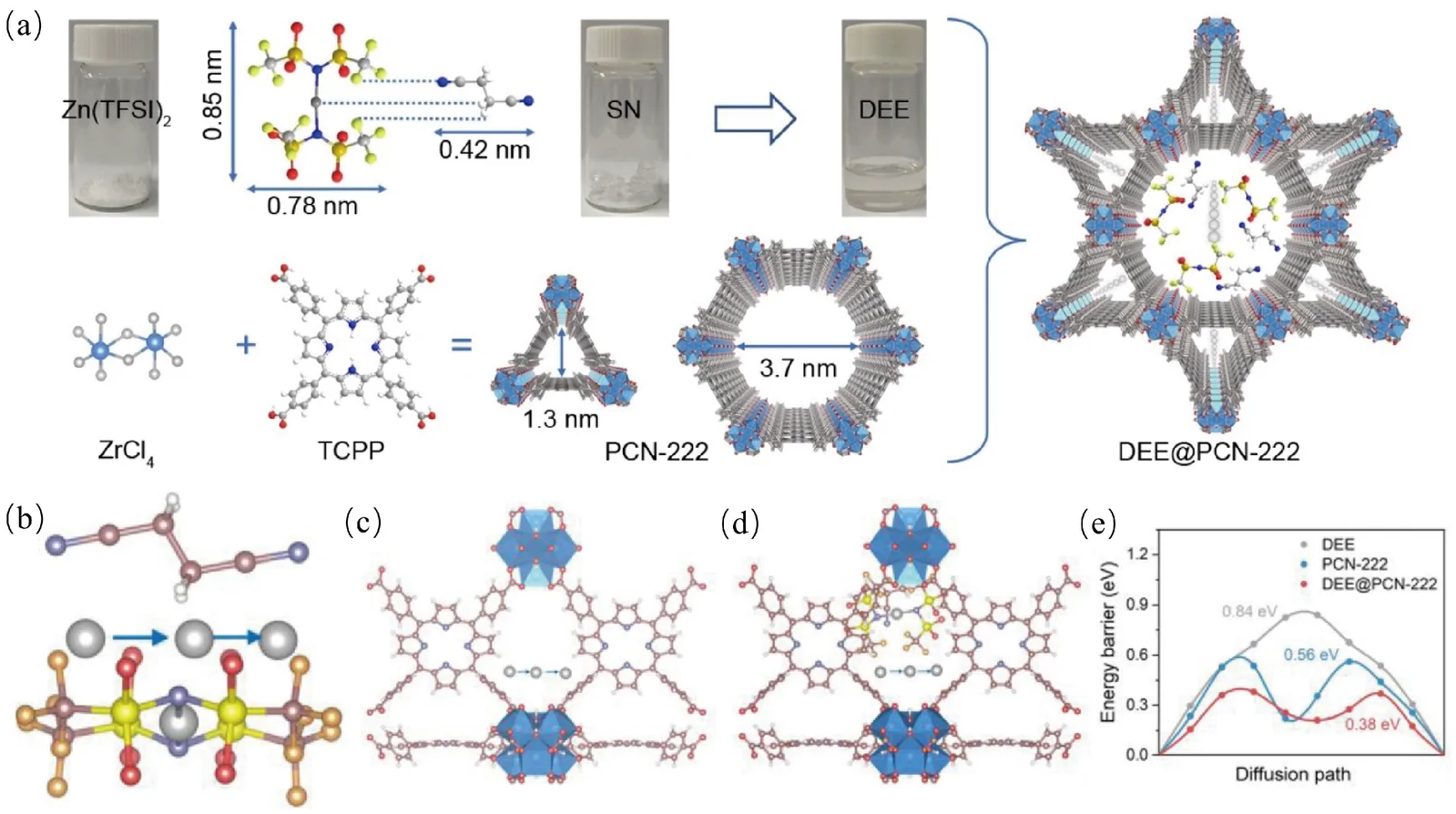
(**a**) Schematic illustration of the overall preparation process of DEE@PCN-222. First-principles calculations of the optimum Zn^2+^ migration pathway in (**b**) DEE, (**c**) PCN-222, and (**d**) DEE@PCN-222. (**e**) The corresponding migration energy barriers (reproduced with permission from [95]).

Gel electrolytes represent a fundamentally different physicochemical paradigm: water molecules and salts are immobilized within the nanopores of a three-dimensional polymer network. Three water states were finely distinguished through DSC—through superhydrophilic group engineering, non-freezable bound water was increased from 0.129 to >0.4 mg mg^−1^ and free water was reduced from 1.232 to ~0.15 mg mg^−1^—achieving 3.25 V ESW at low salt concentration [37]. Hao et al.’s WIPSE anchors water molecules with PAM amide groups, achieving 2.7 V and 3.7 V ESW at 4.1 m and 7.6 m LiTFSI, respectively—matching 21 m and 40 m saturated WiSE—while simultaneously achieving 80% LiTFSI recyclability [62]. Miao et al.’s MOF-confined DEE solid-state electrolyte encapsulates DEE within PCN-222 nanopores through host–guest interaction (Figure 10a). First-principles calculations of the Zn^2+^ migration pathway showed that confinement within the MOF channels shortens the distance between zincophilic sites, lowering the migration barrier from 0.84 eV (DEE) and 0.56 eV (PCN-222) to 0.38 eV (DEE@PCN-222) (Figure 10b–e) [95]. A “lean water gel” regime (5–10 wt% H_2_O) where ion conduction may transition from vehicular to Grotthuss/hopping mechanism is a conspicuous blank on the map.

### 5.3. Asymmetric/Decoupled Electrolyte Design

**Figure 11:**
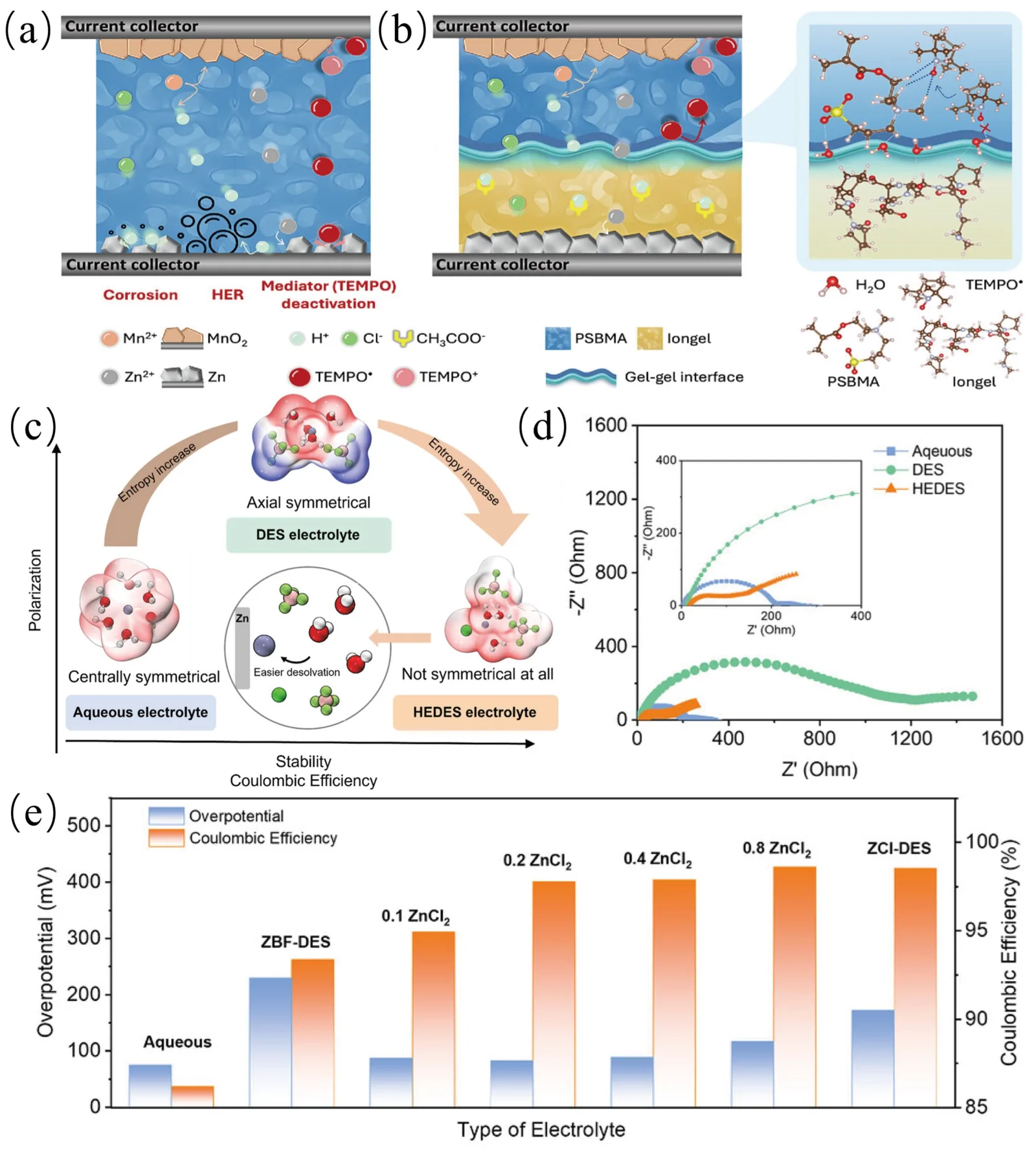
Schematic illustration of the challenges of DEFZMB and the proposed dissimilar electrolyte architecture: (**a**) Conventional homogeneous electrolyte, (**b**) dissimilar electrolyte [59]. (**c**) Diagram of the design idea and solvation structure changes with entropy increase. (**d**) Comparison of overpotential (taken from the fifth cycle) and Coulombic efficiency (average of 50 cycles) test results of different electrolytes. (**e**) Overpotential of DES and HEDES at different cycle numbers [63]. Reproduced with permission from [59,63].

All aforementioned strategies share an implicit symmetry assumption: the same electrolyte simultaneously contacts both cathode and anode. In aqueous batteries, this assumption constitutes a fundamental contradiction; HER suppression favors neutral/alkaline low-water-activity environments, whereas MnO_2_ deposition favors acidic high-proton-activity environments. Decoupling strategies were systematically classified into membrane decoupling, redox targeting, and phase interface configurations [96]. A core-shell solvation electrolyte with asymmetric methylurea molecules was constructed, achieving 4.5 V ESW with in situ DEMS confirming zero H_2_ evolution at 0.5 V vs. Li^+^/Li [97]. Device-level decoupling of Zn–MnO_2_ dual-electrode-free batteries was achieved with heterogeneous gel electrolytes—one self-assembled nano-water film serves as a selective ion transport “soft wall,” achieving 3.1 mWh cm^−2^ areal energy density under lean electrolyte conditions (Figure 11a,b) [59]. Membrane chemical stability, cross-contamination, and the high cost of commercial Nafion membranes (~$500 m^−2^) constitute three fundamental challenges.

### 5.4. High-Entropy Electrolytes: An Emerging Paradigm

The high-entropy concept originates from metallurgy; when five or more elements are mixed in equimolar ratios, extremely high configurational entropy suppresses phase separation and stabilizes single-phase solid solutions. Migrating this concept to electrolyte systems, near-equimolar mixing of multiple salts or co-solvents homogenizes Li^+^/Zn^2+^ solvation environments through maximized configurational entropy. High-entropy DES was first applied to aqueous Zn batteries, using five H-bond donors in equimolar ratios to dilute any single donor–donor strong interaction. Polarization voltage dropped to less than half that of conventional binary DES. Figure 11c shows the solvation structure change from ordered low-entropy to disordered high-entropy. Figure 11d provides hard data directly answering whether high-entropy design truly solves the DES polarization bottleneck. Figure 11e shows DES overpotential starting extremely high, decreasing somewhat but then rebounding due to side-reaction accumulation; HEDES remains below 100 mV throughout, without rebound [63]. A fundamental open question remains: distinguishing genuine “high-entropy effects” from “cocktail effects” (via non-equimolar low-entropy and medium-entropy controls), a distinction recently formalized on the cathode side by Dong et al., who separated configurational-entropy stabilization, lattice hysteresis, and the cocktail effect [98].

### 5.5. Low- and High-Temperature Performance

Low-temperature operation is not an optimization metric but an entry threshold for outdoor energy storage systems [99,100,101,102]. The molecular origin of freezing lies in water molecules’ kinetic energy falling below hydrogen-bond constraints, forcing them into the hexagonal ice lattice. Anti-freeze design principles were articulated in a review; all strategies essentially aim to disrupt the continuous water hydrogen-bond network [103].

**Figure 12:**
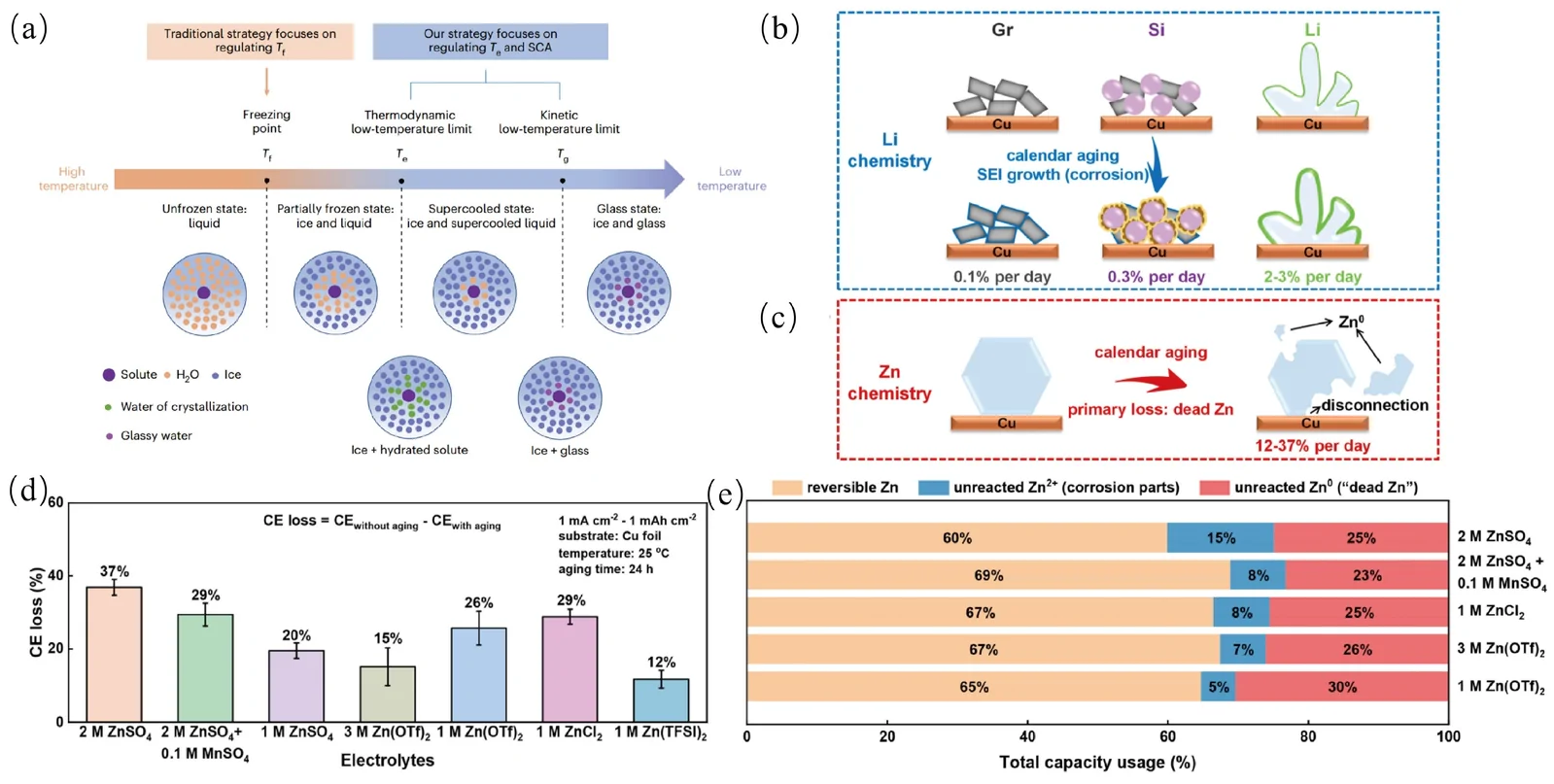
(**a**) Schematic evolution of a dilute solution in the H_2_O–solute system during cooling process and the difference between traditional and our proposed strategies. The freezing process involves several typical temperature terms of H_2_O transitioning to ice (T_f_), H_2_O transitioning to the mixture of ice and water of crystallization (T_e_), H_2_O transitioning to glassy water (T_g_) [69]. Different mechanisms of calendar aging between Li chemistry and Zn chemistry: (**b**) Continuous SEI formation (corrosion) through electrolyte reduction at the anode surface during calendar aging in Li-based battery anodes (e.g., Gr, Si, and Li metal). (**c**) Dead Zn accounts for the large majority of capacity losses during calendar aging in Zn chemistry. (**d**) CE losses of Zn metal in various electrolytes, including 2 M ZnSO_4_, 2 M ZnSO_4_ with 0.1 M MnSO_4_ as additives, 1 M ZnSO_4_, 3 M Zn(OTf)_2_, 1 M Zn(OTf)_2_, 1 M ZnCl_2_, and 1 M Zn(TFSI)_2_ (current density: 1 mA cm^−2^, capacity: 1 mAh cm^−2^, substrate: Cu foil, temperature: 25 °C, aging time: 24 h). (**e**) Quantitative analysis of capacity usage (reversible Zn, unreacted Zn^2+^ (corrosion parts), and unreacted ZnO) in various electrolytes by the TGC method [105]. Reproduced with permission from [69,105].

A landmark three-parameter rational design framework was established. The eutectic temperature (T_e_) is the thermodynamic low-temperature lower bound—the temperature at which the electrolyte completely freezes into a crystalline state. The glass transition temperature (T_g_) is the kinetic low-temperature lower bound applicable only to systems with strong supercooling ability (SCA). Two general design rules are: introduce high ionic potential auxiliary cations (Al^3+^, Ca^2+^) or high donor number co-solvents (ethylene glycol, EG) to construct multi-solute systems, simultaneously achieving low T_e_ and strong SCA. Three Na-based demonstration systems validated the design: Na–H_2_O–Al (T_e_ −53.5 °C), Na–H_2_O–EG (T_e_ −72.6 °C), and Na–H_2_O–Ca (T_g_ −117.1 °C). Full cells still delivered 12.5 Wh kg^−1^ at −85 °C (Figure 12a) [69]. Li et al.’s DMF–KOH electrolyte achieved operation below −82 °C through O–H bond strengthening and alkaline pH [19]. A dedicated review mapped the full landscape of PAM gel anti-freeze strategies; amide groups anchor water as non-freezable bound water that ice crystals cannot recruit [104]. Asymmetric FTFSI^−^ anions were introduced to increase the configurational entropy of the WiSE liquid phase while geometrically hindering ordered lattice formation in the crystalline phase, thereby depressing the salt precipitation temperature by tens of degrees [70].

Beyond the freezing point, the upper temperature bound and, more insidiously, idle time itself impose an equally strict test: calendar aging probes whether the electrolyte and its interphases remain chemically stable when the battery is not cycling at all. The most underestimated failure mode in Zn batteries was revealed through a pioneering study; Zn metal anodes lose 12–37% of capacity after merely 24 h of calendar aging—over an order of magnitude higher than all previously studied battery chemistries. Through titration gas chromatography (TGC), they rigorously distinguished corrosion-induced Zn loss from electrochemically isolated “dead Zn” loss, identifying dead Zn as the dominant contributor to calendar aging—implying a mechanism of localized microbattery spontaneous discharge–redeposition cycles (Figure 12b–e) [105].

High-temperature Zn battery electrolyte failure was analyzed from thermodynamic and kinetic dimensions; HER exchange current density approximately doubles per 10 °C increase. WiSE/WiBS additionally face the special risk of salt precipitation at elevated temperatures. A three-tier countermeasure strategy is as follows: thermally stable salts (Zn(OTf)_2_), heat-resistant interphase-forming additives, and quasi-solid-state systems [106]. The tension between low-temperature (requiring H-bond disruption and low viscosity) and high-temperature (requiring component thermal stability and low reactivity) requirements has yet to be systematically resolved. Temperature-responsive dynamic bifunctional formulations represent a future direction [107,108,109,110].

### 5.6. Cost, Sustainability, and Device-Scale Engineering

The data reported by Xie et al. showing PEG is 30–100× cheaper than LiTFSI implies that salt procurement cost alone for a 1 kWh WiSE battery could reach $50–100 [40]. The OAc^−^ strategy achieves WIS-level CE at ~2 m concentration, directly resolving the cost barrier from the chemical root [74]. The fluorine-free NAG SEI design was explicitly framed within the context of ECHA’s 2023 PFAS legislative proposal; if fluorinated salts are strictly banned or restricted within the next decade, the foundational chemistry of the vast majority of current WiSE/WiBS/DEE/IL research will need to be re-examined [15].

The WIPSE system, first reported by Hao et al., performed the first systematic verification of “electrolyte recyclability” in the aqueous battery field [62]. Ming et al.’s review listed recyclability as one of the four key pillars of aqueous battery full-life-cycle sustainability [111]. A prudent LCA critique of the “aqueous = green” intuition was offered—in the absence of full-chain quantitative data from mining to disposal, aqueous batteries relying on fluorinated salts and high-energy-consumption electrode materials may significantly deviate from this intuition in environmental sustainability [112].

Excess electrolyte (>50 g Ah^−1^), overly thick Zn/Li anodes, and constant 25–30 °C in laboratory coin cells constitute a tight safety net masking the true kinetics of electrolyte consumption and interface evolution [113,114,115]. The 2.5 V LiMn_2_O_4_||Li_4_Ti_5_O_12_ pouch cell by Xu et al. is one of the very few studies to systematically calibrate four critical practical parameters: 2.5 mAh cm^−2^ areal capacity, P/N ratio 1.14, lean electrolyte 3 g Ah^−1^, and 92% capacity retention after 470 cycles [46].

The dual-electrode-free Zn–MnO_2_ configuration proposed by Li et al. pushes device energy density to the theoretical maximum by eliminating the initial Zn anode and MnO_2_ cathode but stringently demands synchronous near-100% CE maintenance on both Zn and Mn sides [47]. The decoupled dual-salt electrolyte (ZnSO_4_ + Zn(ClO_4_)_2_) realized by Li et al. embodies an entirely new design philosophy of “decoupling interfacial chemistry from bulk chemistry”; strongly hydrating SO_4_^2−^ spontaneously enriches at the interface to stabilize interfacial water; weakly hydrating ClO_4_^−^ dominates the bulk, achieving 15.1 mS cm^−1^ high conductivity and 99.97% Zn CE at −40 °C. The pouch cell self-discharge rate is merely 0.13% per day [60]. It was demonstrated that I^−^ selectively adsorbs on Zn(100) and (101) facets, suppressing dendrite growth direction from the earliest nucleation stage and guiding deposition preferentially to the (002) facet; anode-free Zn/I_2_ full cells maintained 88.2% capacity after 10,000 cycles at 50 mA cm^−2^ [61].

## 6. Summary and Outlook

This review is unified by the premise that water’s molecular environment—O–H bond strength, hydrogen-bond topology, and interfacial reactivity—constitutes the central lever for breaking the aqueous battery ESW bottleneck. A common physicochemical language is first established, encompassing the solvation spectrum, hydrogen-bond disruption signatures, EDL dynamics, and dual-mechanism ion transport. The strategy toolkit is then advanced along three progressive dimensions: concentration engineering (anion-shell coordination, though penalized by viscosity, cost, and low-temperature issues), solvation engineering (chemically replacing first-shell water), and interface engineering (SEI/CEI as an irreducible design dimension). Finally, system paradigm innovation—including IL-water/decoupling/gel/high-entropy approaches—completes the toolkit, and every strategy is subjected to practical-utility screening spanning wide-temperature adaptability, calendar aging, cost and PFAS regulation, and coin-to-pouch migration. At the mechanistic level, these strategies can be further understood through three complementary design principles: thermodynamic water activity control, which reduces effective water activity below the HER kinetic threshold via ion-hydration locking (WiSE/hydrate-melt), physical exclusion (crowding/LHCE), or hydrogen-bond competition (DEE/gel); kinetic ion transport and desolvation, which maintains bulk conductivity and low desolvation barriers—through mixed-anion WiBS, LHCE clusters, low-DN co-solvents, or MSE “bridge-activated” recessive solvents—while still suppressing water activity; and proactive interphase construction, where anion-derived SEI, AIL, and CEI repair act as parallel redundancy, providing fault tolerance under electrolyte aging and local inhomogeneity.

Notwithstanding the systematic framework of modulation strategies and mechanistic principles outlined above, which provides a holistic, multi-scale roadmap for breaking the ESW bottleneck in aqueous batteries, the field still faces several unresolved fundamental controversies and critical knowledge gaps. These open issues constrain the rational design of high-performance aqueous electrolytes and hinder the reliable scaling of the technology from lab-scale coin cells to practical industrial applications. Three core controversies remain definitively unresolved. First, the mechanistic origin of ESW expansion in WiSE is still contested between thermodynamic and kinetic interpretations; while McEldrew’s thermodynamic model predicts a substantial reduction in effective water activity, kinetic characterizations consistently yield residual currents far below the values predicted by equilibrium thermodynamics, and the definitive validation via operando Stark spectroscopy has yet to be reported [44]. Second, the long-term structural stability of the aqueous solid-electrolyte interphase (SEI) remains largely unverified; thermodynamic solubility calculations for LiF and ZnF_2_ imply that cumulative dissolution–reprecipitation would degrade the interphase over 10,000 cycles [35], yet nearly all published stability evaluations are limited to fewer than 1000 cycles, leaving long-term cycling durability unconfirmed. Third, the very classification of highly concentrated systems as “aqueous” is increasingly called into question: in a 21 mol kg^−1^ LiTFSI electrolyte, the mass of dissolved salt exceeds that of water by a factor of 2.5 [11,39], situating such compositions much closer to the molten-salt regime on the solution continuum and weakening the core “safe, low-cost, green” value proposition of aqueous battery technology. Beyond these three focal controversies, a broader set of key scientific challenges remains unaddressed, including direct atomic-resolution imaging of solvation structures, operando spatial mapping of interfacial pH distributions, first-principles-driven inverse electrolyte design, and three-dimensional kinetic mapping under lean-electrolyte conditions (electrolyte consumption < 5 g Ah^−1^).

## Data Availability

Data are contained within the article.
